# Model Choice for Quantitative Health Impact Assessment and Modelling: An Expert Consultation and Narrative Literature Review

**DOI:** 10.34172/ijhpm.2023.7103

**Published:** 2023-03-13

**Authors:** Natalie Mueller, Rodrigo Anderle, Nicolai Brachowicz, Helton Graziadei, Simon J. Lloyd, Gabriel de Sampaio Morais, Alberto Pietro Sironi, Karina Gibert, Cathryn Tonne, Mark Nieuwenhuijsen, Davide Rasella

**Affiliations:** ^1^ISGlobal, Barcelona, Spain; ^2^Universitat Pompeu Fabra (UPF), Barcelona, Spain; ^3^CIBER Epidemiología y Salud Pública (CIBERESP), Madrid, Spain; ^4^Institute of Collective Health (ISC), Federal University of Bahia (UFBA), Salvador, Brazil; ^5^School of Applied Mathematics, Getulio Vargas Foundation, Rio de Janeiro, Brazil; ^6^Intelligent Data Science and Artificial Intelligence Research Center, Universitat Politècnica de Catalunya (IDEAI-UPC), Barcelona, Spain; ^7^Hospital Clínic—Universitat de Barcelona, Barcelona, Spain

**Keywords:** Health Impact Assessmen, Ex-Ante Impact Evaluation, Forecast, Modelling, Policy

## Abstract

**Background:** Health impact assessment (HIA) is a widely used process that aims to identify the health impacts, positive or negative, of a policy or intervention that is not necessarily placed in the health sector. Most HIAs are done prospectively and aim to forecast expected health impacts under assumed policy implementation. HIAs may quantitatively and/ or qualitatively assess health impacts, with this study focusing on the former. A variety of quantitative modelling methods exist that are used for forecasting health impacts, however, they differ in application area, data requirements, assumptions, risk modelling, complexities, limitations, strengths, and comprehensibility. We reviewed relevant models, so as to provide public health researchers with considerations for HIA model choice.

**Methods:** Based on an HIA expert consultation, combined with a narrative literature review, we identified the most relevant models that can be used for health impact forecasting. We narratively and comparatively reviewed the models, according to their fields of application, their configuration and purposes, counterfactual scenarios, underlying assumptions, health risk modelling, limitations and strengths.

**Results:** Seven relevant models for health impacts forecasting were identified, consisting of (*i*) comparative risk assessment (CRA), (*ii*) time series analysis (TSA), (*iii*) compartmental models (CMs), (*iv*) structural models (SMs), (*v*) agent-based models (ABMs), (*vi*) microsimulations (MS), and (*vii*) artificial intelligence (AI)/machine learning (ML). These models represent a variety in approaches and vary in the fields of HIA application, complexity and comprehensibility. We provide a set of criteria for HIA model choice. Researchers must consider that model input assumptions match the available data and parameter structures, the available resources, and that model outputs match the research question, meet expectations and are comprehensible to end-users.

**Conclusion:** The reviewed models have specific characteristics, related to available data and parameter structures, computational implementation, interpretation and comprehensibility, which the researcher should critically consider before HIA model choice.

## Introduction

 Health impact assessment (HIA) is a widely-used process for identifying the health impacts – both, negative and positive of a policy, program, project or other kind of intervention. HIA is a tool that can help foresee how a *counterfactual* may affect population health, by systematically assessing associated health benefits and risks. The counterfactual accounts for how a change in health risk factor exposure distribution under a proposed policy, program or intervention may change a population’s health status in comparison to the business as usual (BAU) or other reference situation.^1^ The World Health Organization (WHO) defines HIA as a *combination of procedures, methods and tools by which a policy, program, or project may be judged as to its potential effects on the health of a population, and the distribution of its effects within the population,*^2^ and thereby provides a flexible HIA definition.

 HIAs can be carried out prospectively (ie, ex-ante), concurrently or retrospectively (ie, ex-post).^3^ However, as implied by ‘assessment’ in contrast to ‘evaluation,’ most HIAs are carried-out prospectively to compare health consequences under contrasting futures.^4^ In the context of this article, we will also understand HIA as a tool for forecasting policy proposals, whereas a comprehensive body of literature exists on health impact evaluation (ie, ex-post assessments).^5^

 Societies usually give considerable weight to their self-interest in health,^6,7^ meaning HIAs can generate awareness and have a bearing on the policy decision-making process.^4^ HIA methods can be qualitative or quantitative in nature. Both methods can co-exist and profit from each other.^8^ Qualitative HIA approaches usually draw on evidence that is already available, hence can be carried-out more rapidly and thereby might be less resource-intensive, but they may be more prone to selective and subjective reporting.^3,9,10^ Quantitative HIA approaches numerically assess the magnitude and direction of the expected health impacts of a proposed policy; this means they may be of special importance for influencing policy, as decision-makers commonly give more weight to outcomes that are measurable.^3^ Quantitative HIA is, unlike to qualitative HIA, less sensitive to societal opinions and notions, but can usually only consider a selection of health risk factors of interest.

 A variety of quantitative HIA models have been used to forecast health impacts. However, each of these models comprises a different HIA application area, data requirements, complexities, assumptions, health risk modelling, limitations, strengths and comprehensibility. Thus, the objective of this study was to review the most relevant models for prospective HIA, in order to provide systematic guidance on model choice for future HIA studies.

## Methods

 To review the models, we combined consultation of HIA experts with a narrative literature review. The lead and last author of this manuscript (NM and DR) jointly identified established experts working on quantitative HIA in various fields, including environmental and social epidemiology, econometrics, climate change impact modelling, public health, computer and data science, and invited them to contribute to this interdisciplinary, open discussion on health impact modelling. For ease of understanding, it was decided that this review should cover the broad field of quantitative HIA models. Each expert had worked with at least one of the included HIA models in the past, in various fields of application, including urban planning, transport or environmental sciences, fiscal policies, health care systems research, and infectious disease and non-communicable disease (NCD) modelling, covering low-, middle- and high-income policy settings.

 The narrative literature review complemented the expert inputs, in order to provide a comprehensive overview and model examples. Thus, we did not intend to provide a systematic review of the literature, nor do we attempt to outline all the conceptual and computational steps of HIA models. Rather we aim to provide public health researchers with insights into the broad field of prospective HIA models, their key features and implications, in a form that may serve as basis for model choice. Below, we review each model individually and relate them to quantitative HIA application. We acknowledge, however, that models are often combined, integrated and hybrid-models exist, and models cannot always be as easily distinguished as we do in this review.

###  Key Health Impact Modelling Issues 

 Available data, their respective structures, and the context of the assessment are of high relevance to HIA model choice. From the outset of model development, researchers should work with the end-users (eg, communities affected by the respective policies and their advocates, policy-makers, practitioners, etc), so as to ensure that the questions asked and analysis performed are feasible, relevant, comprehensible, timely and resource-efficient, while mutual expectations are met.^8^ In this regard, there is value in qualitative methods that complement the quantitative implementation of HIA models, as there is a considerable body of literature that stresses the importance of citizens’ participation in planning and decision-making processes,^11,12^ and an increased need for participatory, quantitative HIA implementation has previously been identified.^8^

###  Model Comparison

 To comparatively review the models in terms of application potential for HIA, we developed a data extraction tool based on eight criteria (Table). The criteria and their definitions are:

1) Configuration: Core model functioning and unit.
2) Counterfactual scenario: Definition of scenario construct and comparative elements (ie, BAU with policy scenario comparison).
3) Objectives/purpose: Defines how model outcomes should be treated and interpreted.
4) Assumptions: Underlying assumptions regarding policy effects, population and disease characteristics, data structures and parameters.
5) Health risk function: Health risk modelling or risk extrapolation to establish associations between exposure level changes due to policy effect and health outcomes.
6) Complexity: Model construct and implementation complexity, linear versus dynamic, consideration of heterogeneity versus homogeneity, consideration of covariates, interactions, adaptation, etc.
7) Limitations: Model prerequisites and limits.
8) Strengths: Model strengths and advantages.

**Table:** Analytical Models for Forecasting of Health Impacts in Health Impact Assessment

**Model **	**Configuration**	**Counterfactual Scenario**	**Objective/Purpose**	**Assumptions**	**Health Risk Function**	**Complexity**	**Limitations**	**Strengths**
CRA	• Comparison of BAU vs counterfactual (typically non-healthcare policy), to assess typically NCD impacts • Model unit: population-level	• Comparison of health impacts based on assumed change in health risk factors exposure levels associated with policy	• Standardize and compare health risks and benefits resulting from the change in health risk factors exposure levels • Provide net health impact of policy effect	• Non-temporal model, compares two scenarios with and without change in exposure levels • Frequent assumption of immediate, perfect implementation of policy and build-up of health impacts	• Risk extrapolation from literature, no risk modelling	• Change in exposure level leads to change in health status, static, unidirectional, no feedback • Limited consideration of population heterogeneity and covariates	• Questionable generalizability of risk extrapolation across populations • Exclusive consideration of previously quantified health risk factors • Cannot account for demographic or disease burden changes over time • Limited accounting for competing causes of disease	• Simple but robust model if risk generalizability across populations holds true • High comprehensibility by end-users • Resource-efficient (time, money, computational skills)
TSA	• Considers the ordered sequence of values of a variable (health outcome) at equally spaced time intervals and assumes past values of sequence to be good predictor of future values • Model unit: population-level	• Prediction of post-intervention period under assumption that intervention would not have been implemented	• Forecasting future values of sequence based on historical trajectory • Comparison of regression slopes of implemented policy vs BAU (retrospective/ex-post HIA)	• Past values of variable are predictors of future values • Time is independent • Assumptions of autocorrelation (data points have internal structure), seasonality (periodic fluctuations), stationarity (constant mean and variance)	• (Univariate) regression of future values with set of past values • No consideration of covariates, except dynamic regression models • Researcher chooses correlation structure	• Mostly static, forecast of future values based on past values, all other parameters held constant • Limited consideration of population heterogeneity and covariates • Unidirectional, no feedback	• Availability of historic data • Reliability depends on correctly identifying and accounting for time trends • Health outcomes are rarely stationary • Structural breaks lead to instability • Retrospective/ex-post HIA only as policy is implemented	• Robust model if detailed time-series data is available and past is indeed good predictor of future • Resource-efficient (time, money, computational skills)
CM	• Individuals are assigned to health-state compartments, they progress along • Used to estimate health-state transitions for infectious/NCD developments and how policies may change these transitions • Model unit: population-level	• Comparison of BAU vs policy scenario and changed health-state transition probabilities	• Forecast infectious/NCD evolution, considering disease and population specific health-state transition probabilities	• Compartmental applications (aggregation of individuals into health states) • Individuals in each compartment have same transition probability	• System of ordinary differential equations • Mostly risk extrapolation from literature, but also risk modelling (researcher decides on model parameters)	• Models range from simple to complex, depending on number and relationships between compartments, consideration of heterogenous population and disease characteristics	• Same health state transition probability for all individuals (population proportions) in the same compartment • Different models for the same infectious/NCD, depending on which population and disease-specific characteristics are considered	• Robust model for compartmental disease and population characteristic transitions • Basic models are resource-efficient (time, money, computational skills)
SM	• Forecasts health impacts using regression analysis, considering interdependencies of endogenous and exogenous factors on causal pathway • Model unit: population-level	• Comparison BAU vs policy scenario and changing endogenous and exogenous parameters to represent policy effects under varying conditions	• Forecasts policy impacts under different conditions and environments, considering interdependencies of endogenous and exogenous factors on causal pathway	• Policy effect differs according to different conditions and environments studied • Causal interdependencies of varying endogenous and exogenous factors lead to differential policy effects	• Multivariate regression analysis used for prediction • Change of parameters to represent policy effect • Risk modelling (researcher decides on model parameters)	• Functional forms of equations affect model estimation • Need for good data- and theory-driven insight into interdependencies of endogenous and exogenous factors on causal pathway	• Researcher decides on model parameters, which influences results, as it is impossible to capture every endogenous and exogenous factor of reality (limited system) • Fully-specified models needed to reduce uncertainty • Resource-intensive (time, money, computational skills)	• Considers varying effects under varying conditions and environments (interdependencies of endogenous and exogenous factors) • Goes beyond conclusions of conventional empirical studies that provide reduced-form causal relationships
ABM	• A set of agents with defined attributes on the micro-scale interact with and adapt to each other and their environment over space and time, and produce social phenomenon on macro-scale • Model unit: individual-level	• Comparison of BAU vs policy-induced changes in the environment and/or agent’s properties, rules actions and interactions	• Bridging individual-level assumptions and population-level dynamics and thereby exemplifying a complex social phenomenon • Uncover adaptive system responses that can help understand the long-term policy impact	• Researchers chooses agent’s properties, rules, actions, time and environment and thereby causal mechanisms • Simplification of the real-world, best if data and theory-driven	• Causation via a social phenomenon • Computational model • Risk extrapolation from literature, or risk modelling (researcher decides on model parameters)	• Highly complex (modeling agent interaction, adaptation and feedbacks) • Detailed individual-level data required	• Sensitivity to modeling decisions made by researcher • Potential lack of transparency on causal pathways and uncertainties involved • Lack of real-world data for model calibration and validation • Potentially limited comprehensibility by end-user • Resource-intensive (time, money, computational skills)	• Complex system insight into causal mechanisms and adaptive system responses • Consideration of agent heterogeneity (properties, rules, actions), interaction and adaptation • Useful to study social phenomenon that arises from changing dynamics at micro-level
MS	• Individuals’ trajectories are simulated, with transitions between states and conditions over time, but with no interaction and adaptation • Model unit: individual-level	• Comparison BAU vs policy-induced changes of individuals’ trajectories	• Provide detailed policy impact for individuals or subgroups and thereby gain insight into equity implications	• All unit-level parameters need to be known or are reasonably imputable	• Multivariate regression analysis used for prediction • Risk extrapolation from literature, or risk modelling (researcher decides on model parameters)	• Highly complex (modeling individuals’ trajectories) • Detailed individual-level data required	• Difficult to explain in detail • Potentially limited comprehensibility by end-user • Resource-intensive (time, money, computational skills)	• Individuals’ trajectory insight • Consideration of population heterogeneity (policy effects and health outcomes)
AI/ML	• Computer algorithms recognize patterns in complex and changing data and learn from them • Model unit: individual-level	• Supervised learning predicts a particular social phenomenon by discovering relevant patterns in data	• Algorithm is trained to detect pattern in data and can detect them under changing data structures	• A set of rules is applied to the algorithm that leads to the recognition of the desired pattern and the algorithm evolves by learning from it	• Computational model • Risk modelling (algorithm decides on simulation parameters and functional forms)	• Highly complex (algorithm development)	• Quickly-evolving field with no standards defined • Difficult to explain in detail • Overfitting of data limits forecasting performance • Limited comprehensibility by end-user • Resource-intensive (time, money, computational skills)	• Insight into complex, real-world non-linear associations under changing data and conditions

Abbreviations: ABM, agent-based model; AI, artificial intelligence; HIA, health impact assessment; BAU, business as usual; CM, compartmental model; CRA, comparative risk assessment; ML, machine learning; MS, microsimulations; SM, structural model; TSA, time series analysis; NCD, non-communicable disease.

**Figure 1:**
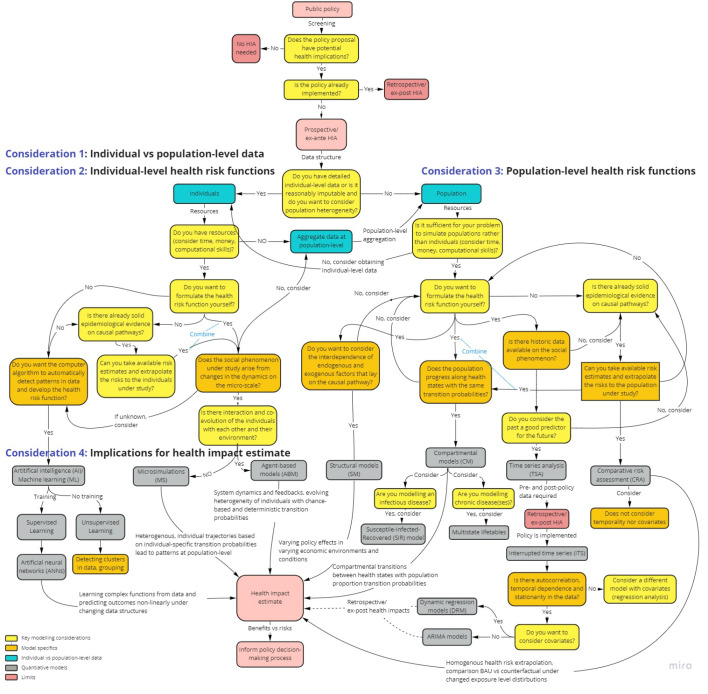
Flowchart for Health Impact Assessment Model Choice Considerations. Abbreviations: HIA, health impact assessment; BAU, business as usual.

 We also developed a flowchart (Figure 1) intended to guide researchers in their HIA model choice, considering the research question and field of HIA application, available data structures and resources, model functioning and specifics, and interpretation of model outputs.

## Results

 We identified seven HIA models, that have been used or have the potential to forecast health impacts of policy proposals. We comparatively summarized the models in Table. Moreover, Figure 1 provides key considerations for HIA model choice.

 The seven reviewed models are: (*i*) comparative risk assessment (CRA), (*ii*) time series analysis (TSA), (*iii*) compartmental model (CM), (*iv*) structural model (SM), (*v*) agent-based model (ABM), (*vi*) microsimulations (MS), and (*vii*) artificial intelligence (AI)/machine learning (ML).

###  Comparative Risk Assessment

 In CRA the health impact due to an observed health risk factor exposure distribution in a population (ie, BAU) is compared with the health impact under a hypothetical health risk factor exposure distribution (ie, counterfactual), representing the policy proposal.^13^ CRA aim to provide a standardized and comparable estimate of the expected health impact of shifting from the BAU to the counterfactual scenario, and the distribution thereof among the population.^13,14^ CRA, in comparison to the other models, should be understood as a comparison of BAU with an alternative “future” scenario, and therefore, CRAs are more static and can only restrictedly consider temporal elements such as an aging population. CRAs typically deal with long-term exposure and NCD outcomes (eg, changes in premature deaths, cases of NCDs),^13^ can consider multiple health risk factors and aim to provide a net health impact (ie, positive or negative) of the policy proposal.^13,15^ Steps in CRA are commonly (*a*) definition of counterfactual (ie, policy proposal); (*b*) identification of health risk factors of interest; (*c*) estimation of population health risk factor exposure distribution; (*d*) selection of health outcomes of interest; (*e*) selection of exposure-response functions (ERFs) from the scientific literature to quantify the association between the health risk factor exposure level and the health outcome; (*f*) combination of health risk factor exposure data and ERFs for each population subgroup under consideration; (*g*) quantification of the magnitude of the expected distribution of health impact in the population, typically via potential impact fractions; (*h*) quantification of uncertainty and provision of the range of the potential effect.^16^

####  Application in Health Impact Assessment

 CRA is frequently used in HIA studies, with the counterfactual scenario representing the policy proposal (often non-healthcare) under study, and therefore an alternative “future.” Common assumptions are immediate and perfect implementation of the policy, as well as homogenous uptake and unfolding of policy effects among the population. CRA typically draws on health risk functions (ie, ERFs) from previous studies on the assumption that they are generalizable to the study population. Typically, simple CRAs are static and cross-sectional, meaning they simply compare BAU with an alternative health risk factor exposure distribution. They hold all population parameters constant, do not consider potential varying health risks for subgroups, and assume unidirectional relations from cause to effect (ie, no feedback).

 Common limitations include a selective consideration of previously quantified health risk factors and disregard of potentially important factors with weak evidence base; limited consideration of interactive, synergetic effects of multiple health risk factors, leading to potential double-counting of health impacts^17^; lack of temporal considerations (eg, changing population structure over time) and inability to account for competing causes of disease^18^; frequent assumption of no time-lags in health impacts (ie, immediate impacts); the lack of consideration of required individuals’ behavior change^19^; limited consideration of benefit-risk trade-off distortion when comparing long-term benefits with immediate risks (eg, long-term health benefits of physical activity compared to the immediate risk of suffering a traffic accident, related to a policy that promotes cycling for transport^19^); questionable generalizability of health risk functions (ie, ERFs) as they are extrapolated from another population^20^ and capture the population rather than individual-level.

 Despite its uncertainties and limitations, strengths of CRAs are that they can be carried out relatively rapidly, are less data and resource intensive than other HIA models (eg, models that estimate individuals’ trajectories, such as ABM or MS), and provide an evidence-based, health net impact that can be used to inform the policy decision-making process with a typically high comprehensibility for end-users (eg, communities affected by the policy and their advocates, policy-makers, practitioners, etc). CRAs have been widely used, for instance, to model comparatively a wide range of health risk factors within the Global Burden of Disease study,^21,22^ or more specifically, to model city-specific the health impacts of urban and transport planning interventions.^23-25^

###  Time Series Analysis

 Time series data refers to a time sequence of observations of a certain variable, usually at equal time intervals and for a single unit of analysis, eg, an individual or a geographic region, etc. TSA typically draws on the assumption that the past is a good predictor of the future. Several TSA models are available to forecast health outcomes. Considering the nature of the outcome variable, temporal dependence, and covariates, guide in selecting the most suitable model.

 The autoregressive (AR) model assumes the value of a variable at a given moment is dependent on its value at previous time points (ie, autocorrelation), and regresses the current value with a set of past observations of the sequence. The autoregressive moving average (ARMA) model additionally introduces temporal dependence of the random errors, meaning the assumption that the next observation is the mean of all past observations. The AR and ARMA models both rely on the assumption of stationarity, which implies that the mean and variance are constant over time (ie, non-seasonal, and when the time-series fluctuates, it does so uniformly around a particular time). Since the stationarity assumption is commonly unrealistic in health outcomes, as they usually have a seasonal component, the ARIMA (autoregressive integrated moving average) model is preferred, which accommodates non-stationary behaviors in time-series.^26^ ARIMA models achieve stationarity by taking a series of differences, ie, measuring how many non-seasonal differences are needed to achieve stationarity. As usual in statistical modelling, the ARIMA model consists of identification, estimation and application. During the identification step, autocorrelation and partial autocorrelation functions check seasonality, and other trends in the series. These steps allow to find the appropriate linear model for which parameters are estimated through the maximum likelihood estimation. Finally, the model is applied to make forecasts for future points in time.

####  Application in Health Impact Assessment

 TSA are substantially different from other HIA forecasting models, because they can only predict, based on historic data, what would have happened if the counterfactual (ie, policy) would not have been implemented. Extended TSA models can overcome this limitation: The interrupted time series (which may be AR, ARMA, or ARIMA), is a quasi-experimental method used to estimate the impact of a policy implemented at given moment over a single time-series. Interrupted time series, however, do not allow the inclusion of covariates; that is, traditional ARIMA models assume an outcome depends only on its past levels. In contrast, regression models can consider the influence of covariates but not the temporal dependence of the time-series. Dynamic regression models, which may be considered extended ARIMA models, overcome these limitations and can simultaneously include covariates and account for temporal dependence,^27,28^ to give a flexible forecast of the post-intervention period.

 Nevertheless, the above approaches require multiple observations that are taken pre- and post-policy to robustly assess the impact, and are therefore mostly applied in retrospective HIA. The effect of the policy is evaluated by comparing differences in the regression slopes – that is, the difference in the trajectory of the health outcome of interest under the counterfactual (ie, policy implemented) compared to the BAU. However, TSA may be used for forecasting if the historic trajectory is known and the researcher has an idea of how a policy may change this trajectory (ie, the change in regression slopes). Examples of retrospective HIA using TSA include modeling the impact of an Australian health policy restricting the conditions under which an antipsychotic medicine could be subsidized, predicting the number of monthly drug dispensings,^29^ or assessing mortality impacts of air quality changes due to a Hong Kong shipping emission policy.^30^

###  Compartmental Models 

 CMs are typically used for modelling infectious diseases but may also be used to model NCDs. The main CM assumption is that the individuals composing the study population can be aggregated into (mutually-exclusive) compartments, according to their health status, and proportionally move from one compartment to another, as their health status changes. CMs are usually run with a system of ordinary differential equations, and may be deterministic or stochastic. One of the simplest models in infectious disease research is the Susceptible-Infected-Recovered model. In the model, all individuals are susceptible to the disease at the begin of the epidemic and there are one or more initially infected cases. The progression from S to I, at each time step, is usually defined by the product of the number of susceptible individuals, the number of infected individuals, and the effective contact rate (ie, the proportion of contacts that transmit the disease). The recovery rate – the percentage of infected individuals that recover at each time step – is another fundamental parameter in a Susceptible-Infected-Recovered model. More sophisticated CM models are typically used, being customized to the infectious disease and population of interest, considering demographics or other heterogenous population characteristics. As a result, the same disease can be modeled through different CMs, depending on which parameters are included in the model.^31^

 For NCDs, various CMs exist. For instance, the RIVM Chronic Disease Model represents 28 NCDs with compartmental transitions occurring due to changes in risk factor classes, incidence, remission, disease progress, and mortality.^32^ Also, multistate lifetables can generally be understood as CMs as well, as they follow a population experiencing state transitions over time as they age, given changing transition rates and probabilities,^33^ and (often) the simplifying assumption that diseases occur independently of one another.^18^ The proportional multistate lifetable allows to model for multiple diseases as proportions of the population can co-exist in more than one compartment, which relaxes the assumption that compartments are mutually-exclusive.^34^

####  Application in Health Impact Assessment

 CMs have been widely used to assess infectious disease interventions, including both, vaccination strategies,^35^ and non-pharmaceutical interventions, such as wearing face masks for preventing the spread of COVID-19.^36^ NCD intervention strategies have also been assessed, where the counterfactual, ie, the proposed policy, generally represents changed transition probabilities relative to BAU. For instance, multistate lifetables have been used to model health outcomes of policies targeting modifiable health risk factors, such as physical activity or obesity.^18^

###  Structural Models 

 SMs consider how a specific (health) outcome relates to relevant exogenous factors in the specific environment studied, ie, the potential interactions and interdependencies of a policy with other existing policies (macro-level), and endogenous factors, ie, individual characteristics and behaviors (micro-level).^37^ Therefore, SMs provide a framework for understanding how changes in health outcome might relate to distinct environments and conditions.

 The researcher needs to define the variable the model shall compute (ie, health outcome), and which endogenous and exogenous variables to include based on the structural connections between them. There may be multiple plausible models that fit the data equally well. Therefore, the researcher needs to look at the satisfying description of the behavior of the included variables, by checking the available data against theory. Hence, the researcher needs to define a set of formulations with unknown parameters, compute for each formulation the values which give the best explanation of past evolutions, using as criteria statistical testing and compliance to theory. The model estimation can call for the introduction of new variables, or changes in their definitions. Once the model is satisfactorily estimated it can be used for forecasting. SMs can rely on linear methods, but more typically SMs are composed of non-linear estimations and dynamic stochastic models.^38^

####  Application in Health Impact Assessment

 SMs can be used to forecast policy impacts, by drawing on the BAU estimated regression models and changing their parameters to represent the policy, and its impacts on the interdependencies of the endogenous and exogenous factors in the model.^5,39^ Unlike reduced-form estimations, which consider a one-way relationship between an intervention and an outcome, SMs explicitly specify interdependencies between endogenous and exogenous variables, and therefore, provide insight into indirect effects, mechanisms and factors that lay on the causal pathway.^40^ SMs are widely used in econometrics, however, are increasingly being used in health research. For instance, SMs have been applied to forecast the impacts of fiscal policies (eg, restrictions or expansions of fund transfers from government to municipalities) on different health indicators in Brazil (eg, infant mortality, antenatal care visits, access to primary care, premature mortality).^41^

###  Agent-Based Models

 ABMs are simulation models that may predict the appearance of a macro-scale social phenomenon, that arises from changing the dynamics on the micro-scale (ie, agents’ behaviors, actions and interactions in a system).^42^ In ABMs, agents are autonomous individuals/units that behave according to given decision-making rules, within a specified institutional environment (eg, under certain policies), and in defined-interactions, including feedback-interactions. In contrast to other models, ABMs allow agents to adapt (either biologically or behavioral via learning) to each other and their environment over time.^43^ By explicitly modelling every individual, ABMs have the advantage that no population-level aggregation is needed, allowing for representation of real-world heterogeneity of individuals (eg, biological, behavioral, demographic diversity, etc). Moreover, ABMs can take different spatial structures across space and time, which are difficult to represent with other models that rely on mean-field approximations and aggregation.^43^

 The different elements in ABMs can be organized according to the “Properties, Actions, Rules, Time, Environment” framework.^43^ Properties, actions and rules define the agents, while time and environment define the context. Properties are individual characteristics of the agents, actions define the specific behavior of the agents and can lead to a change in the agent’s own properties or rules, the properties of other agents, and the environment. For every action included, the researcher must define rules under which the action is triggered, and each action must have consequences. Rules define how agents choose actions, change properties and interact with each other and their environment. Time is the unit in which change in rules, actions, properties or environment are defined. The environment provides the boundaries and context for the agents and their interactions and can change over time as a result of agent action or as a result of an intervention policy.

 In ABMs, events occur either by chance, according to probability distributions defined by the researcher, or deterministic behavioral rules not subject to chance.^44^ Hence, in ABMs some elements remain beyond empirical observation, while other elements are statistically estimated,^44^ for instance, by drawing and extrapolating health risk functions from the literature to the agents based on their individual exposure and risk.

 ABMs are complex and flexible to work with, which is part of their power and at the same time limits them. Assumptions on properties, actions, rules, time and environment need to be well grounded and are best if data-driven or theory-based. In contrast to other models, ABMs cannot be compactly described by a set of mathematical equations, which makes it difficult to critically assess them. To overcome this, it has become practice to accompany ABMs with “Overview, Design Concepts and Details plus Decision-Making” criteria, which aim to standardize the model description.^45^ Translating an ABM design into computational code is challenging, specific choices of functional form or algorithms can affect the results of an ABM, and one can get lost in translation during the conceptual to computational transition.^43^

####  Application in Health Impact Assessment

 With regards to HIA, ABMs are useful to explore the potential impacts of policies in dynamic social and physical environments, therefore, considering non-linear relations and being performed under more realistic conditions. ABMs can unveil adaptive and potentially diverse responses, and thus, provide insight into differential policy uptake and impacts. ABMs have been applied in the field of social epidemiology, eg, modelling the associations between gender, socio-economic status and smoking behavior,^46^ and income inequalities, residential segregation and dietary intake.^47^ Recently, ABMs have been applied to demonstrate the effect of social distancing (micro-scale) on reducing the spread of COVID-19 (macro-scale).^48^

###  Microsimulations

 MS are increasingly being used in public health research because of their ability to evaluate intervention effects considering population heterogeneities.^49^ MS are able to simulate the differential effects of policies across specific subgroups, and therefore also provide insight into health equity implications.^50,51^ The MS modelling process is based on the simulation of each unit (ie, individuals, municipalities, regions, etc) that compose the population under study, according to mutually-exclusive and collectively-exhaustive health states.^52^ In contrast to ABMs, in MS, individual units do not take decision-based actions nor interact.

 MS represent a bottom-up approach to obtain population-level results: MS simulate the trajectories of all units and summarize individual unit results to obtain aggregated population-level insight. In MS, each unit changes its state based on unit-specific transition probabilities, which makes MS models different from CMs where the same transition probability applies to all units in a given compartment. MS transition probabilities can follow the Markov assumption assuming that the transition probability depends only on the current state, can consider the states or values of other demographic and socioeconomic variables of the unit, or other more complex transition rules.^53^ MS models follow a cohort of individuals/units and either estimate health risk functions (ie, ERFs) for this cohort directly, or more commonly take and extrapolate health risk functions from the literature. A useful distinction of MS is between static or dynamic: the static models seek to reproduce the impact of a policy on the units at a given time point, while the dynamic models track unit trajectories and transitions over time.^54^

####  Application in Health Impact Assessment

 MS represent a powerful tool to simulate the impact of policies with a high level of precision not achievable with other models. MS could be considered among the most flexible models to evaluate policies on different population and health outcomes and MS can consider interaction and synergistic effects of multiple health risk factors. However, MS require large amounts of data to holistically represent individuals’ heterogeneity. MS usually require the construction of complex codes that use parallel computing and are time and computationally demanding.^52^ To overcome these barriers, in the context of public health research, some user-friendly platforms have been created to allow policy-makers and practitioners to develop customized HIAs and to facilitate and speed-up the execution of MS,^55^ such as the UK Health Forum microhealth simulation model^56^ and the Organization for Economic Cooperation and Development Strategic Public Health Planning model.^57^ Further examples of MS application in the context of public health research include: estimations of the impact of sugar-sweetened beverage taxation on obesity in India,^58^ the effects of fiscal policies on premature mortality in Scotland,^59^ and recently, the cost-effectiveness of COVID-19 control strategies in South Africa.^60^

###  Artificial Intelligence/Machine Learning 

 AI is a broad scientific discipline, aiming to understand and disentangle complex non-linear systems. Despite being a wide discipline, the most popular areas of AI is ML, which refers to the study of computer algorithms that develop automatically by recognizing patterns in data and learning from them.

 ML is divided in two main areas, supervised and non-supervised learning. Supervised learning tackles predictive problems, with the aim to gain insight into a particular phenomenon, emerging from labeled data observations the algorithm is trained with.^61^ Unsupervised learning solves grouping problems or characterization of interactions between variables, and helps to find unknown patterns in data, with the algorithm trying to learn from underlying data structures.^61^ Two common unsupervised learning tasks are clustering and dimensional reduction. In clustering, the algorithm attempts to group data observations into meaningful clusters (eg, grouping patients with similar disease evolution, etc). In dimensional reduction, the algorithm reduces the number of variables by identifying redundant or noisy attributes and grouping similar or correlated attributes for better model training.

 Among ML models, artificial neural networks (ANNs) have been developed as a computational analogy of brain networks of neurons to learn complex functions from data in order to predict (health) outcomes non-linearly. ANNs can solve problems that would be too complex for classical statistical methods as ANNs have self-learning capabilities that enable them to adapt and produce results as data is changing or more data becomes available. ANNs are built like neuron nodes of the human brain, called processing units, interconnecting a web. The processing units are made up of input and output units. The input units receive various forms of data and based on an internal weighing system and activation functions, the ANN attempts to learn and produce an output report. An ANN initially goes through a training phase where it learns to recognize patterns in data. During this supervision, ANN compares the actual output produced with what it was meant to provide. The difference between both outputs is adjusted by correcting the weights of the connections until the difference between actual and desired outcomes has the lowest possible error.

 In comparison to more traditional statistical methods, ML tends to be free of distributional assumptions that often are violated in real-world applications and they can account for non-linear relationships in a longitudinal manner. Instead of the researcher defining the model parameters, it is the computer that decides which parameters to include to best represent the data and reveal complex patterns. ML algorithms can examine larger data sets and disentangle patterns much quicker than other traditional methods. While freedom of distributional assumptions could be considered a strength, ML methods are limited by the characteristics of the training database by fitting it extremely well (ie, overfitting problem), ignoring potentially important real-world aspects that are not included in the training database.^62,63^

####  Application in Health Impact Assessment

 ML is increasingly being used in public health/HIA research and has been used to predict healthcare outcomes either numerically or qualitatively,^61^ eg, describing diagnostics, by drawing on supervised learning, or to identify units that can be grouped together, eg, patients with similar disease evolution, hospitals with similar structure, by drawing on unsupervised learning. A 2019 example used ANNs to identify individuals at risk of in-hospital mortality.^64^ The model not only allowed researchers to model the probability of death longitudinally, but also was able to document how various timestamps contributed to the prediction. Therefore, the researchers were able to provide targeted interventions, as well as forecast changes in the in-hospital mortality probability over time.

## Discussion

 We reviewed seven models that can be used in HIA modelling, to gain insight into the health impacts of policy proposals. The reviewed models have different configurations, fields of application, assumptions, health risk functions, complexities, limitations and strengths.

**Figure 2:**
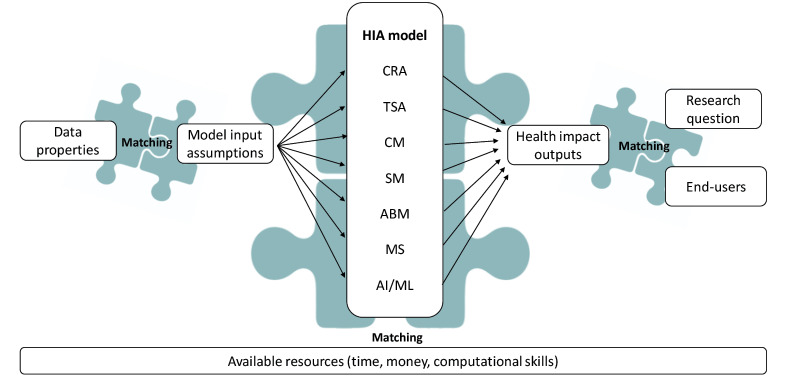
Interlinkages of Key Considerations for Health Impact Assessment Model Choice. The model input assumptions must match the data structure and parameter properties that will be used for model input, while health impact outputs must match the research question to be answered and must be comprehensible to the end-user the HIA is conducted for. Available resources in terms of time, money and computational skills are overarching and decisive. The figure is adapted from Gibert et al.^61^ Abbreviations: HIA, health impact assessment; CRA, comparative risk assessment; TSA, time series analysis; CM, compartmental model; SM, structural model; ABM, agent-based model; MS, microsimulations; AI/ML, artificial intelligence/machine learning.

 Before choosing a model, the researcher needs to consider that model input assumptions match the available parameter and data structures, while the model outputs must match the research question and need to be comprehensible and acceptable to the HIA end-users (eg, communities affected and their advocates, policy-makers, practitioners, etc, while available resources (ie, time, costs, computational skills), are overarching and decisive (Figure 2).^61^

 The research question needs to be defined clearly, best if in consultation with the end-users,^8^ from the outset of the project. The inventory of existing literature and explanatory theory, available data and its structure, and needed team expertise need to be clear. Agreement on research question, timeliness, resources available, and expected use of the outputs between the model conception and design teams and potential end-users are important for model choice and set the boundaries of the work.^61^

 Comparing the seven reviewed models in their structural configurations, the first difference is the modelling unit: while CRA, TSA, CM, and SM model outcomes on the population level, ABM, MS, and ML model individuals and their trajectories, from which eventually population-level patterns derive, ie, social phenomenon under study. The modelling unit, ie, individual vs population-level, together with the complexity of the models, usually determine the amount and variety of input data required, with CRA and TSA usually requiring less data, and ABM and MS the most. However, there is considerable variability and there can be very advanced CRA or simple MS. The structural complexity of the model also determines the requirement of resources and computational skills. ABM, MS, and ML typically demand more advanced mathematical and programming competencies and might require parallel computing, while CRA, TSA, CM, and SM are typically easier implementable. However, with increasing computing power and access to cloud computing, this distinction is less pronounced. If researchers decide to do multiple uncertainty simulations (eg, Monte Carlo) to better quantify the range of potential effect, these can considerably increase model run times.

 All models have the potential for prospective HIA application, except TSA only partially, as TSA models typically require post-intervention observations (unless the change in regression slope is known). CRA outputs should be interpreted as a comparison between BAU and some (future) alternative, as CRA are inherently non-temporal and cannot account for changing population structures, while the other models are simulation approaches and can account for temporal changes and eg, aging populations. While CRA exclusively draw on health risk extrapolation, and therefore assume generalizability of risks to the population under study, all other models can estimate health risk functions, or draw on a combination of risk estimation and risk extrapolation, such as CM, ABM, and MS. While in TSA, CM, SM, ABM, and MS it is the researcher who decides on parameter inclusion for risk estimation, which should be robustly data-driven and theoretically-grounded, in ML it is the algorithm that decides automatically on the model form and function. Hence, ML models are very flexible as they tend to be free of distributional assumptions, eg, variable’s interdependences, higher order interactions, linearity, that the other models depend on, but which are often violated in real-world applications. Nevertheless, ML models are prone to over-fitting, meaning that the model fits extremely well to the training data it was constructed on, but might poorly forecast future values under changed conditions, which might limit ML in HIA application.^63^ This raises generally the question whether extrapolation of available health risk functions from one population to another (as in CRA, and possibly CM, ABM, and MS) are of higher or lower order of validity than obtaining health risk functions from a single dataset with a set of observations in a given population (all other models).

 A key consideration for HIA is timeliness, namely providing evidence of the policy impact *before* its implementation, to influence the decision-making process. Often, a narrow time window is available, after which outcomes are no longer relevant, because a decision on policy implementation has been made. The researcher, according to research question, data structures, end-users and available resources (ie, time, money, computational skills), needs to decide whether to choose a more complex or simpler model and how much added value a more complex model has over a simpler model that achieves similar results. Modelling should follow the principles of parsimony, which refers to preferring simpler models over complex ones, if the simpler model produces robust results. Hence, a simple, comprehensive model, eg, in terms of number of model parameters, distributional assumptions, model form, etc, is preferable over a complex model where the number of parameters is exhaustive and distributional assumptions and model form cannot be understood anymore. However, the robustness of very simple models might be difficult to assess, especially if they try to model a complex social phenomenon. On the other hand, very complex models, without proper description, could be considered as “black boxes” by end-users, running the risk of not being understood and outputs not being used (eg, in the policy decision-making process). Concerns may arise from the choice of formal tools, eg, computer language and modelling packages, because often softwares and algorithms are not standardized, open-source and are continually evolving.

 During the HIA modelling, a multidisciplinary team of experts is desirable, including experts in theory and mechanisms, experts in mathematical modelling and computational coding that help translating the model from concept to code, and experts that can translate the outputs into policy and societal impact. Generally, there is a call for more participatory, quantitative approaches in HIA.^8^ Participation allows planners and policy-makers to gain a more detailed insight into stakeholder behaviors and preferences and is valuable to backup assessment procedures of policy proposals. Moreover, participation allows an increase in public acceptability of decisions, build stronger consensus and reduce conflicts.^8^

 The WHO HIA definition also calls for assessing “*the distribution of [policy] effects within the population,”*^2^ and thereby emphasizes that health impacts of policies can vary for different subgroups, according to varying genetic, lifestyle, or socio-economic, etc, susceptibilities. Studying these differential impacts will help identify most vulnerable subgroups and will pinpoint where intervention is most urgently needed for equitable and just health outcomes. In this regard, individual-level models that allow for identification of subgroup impacts (ie, ABMs, MS, ML) are particularly valuable to understand which individuals/subgroups are disproportionally affected, which is key in defining equitable intervention strategies.

 Several common challenges in health impact forecasting have previously been identified^65^ and need consideration in the health impact modelling discussion. Challenges relate to (*a*) modelling the time course of the counterfactual, ie, immediate policy implementation versus phase-in and the resulting time-varying effects of health drivers on health outcomes; (*b*) time-lags in health benefit build-up and considerations of discounting future impacts; (*c*) uncertainty in addressing structural and parametric uncertainties; (*d*) general uncertainty in projecting the future, related to health drivers, demographic and socioeconomic trends, and health outcomes; and (*e*) how to deal with structural breaks and unforeseen events (eg, pandemics, world economic crisis, etc), leading to instability of model parameters.

 In the context of Georg Box’s reflection of *all models being wrong, but some being useful*, it is important to highlight that all models are limited systems and can only reflect a small fraction of the real-world complex systems. Models are very much tied to the datasets they were constructed on and might miss aspects that are not captured in the data but are important in the real-world. Therefore, having a good theoretical understanding of the social construct under study, including unobserved entities or unmeasured variables, is a prerequisite for model choice and model functioning (eg, structural and parametric robustness). Also, statistical modelling, meaning the fitting of the model to the underlying observational dataset (ie, empirical analysis), should be done complementary to the simulation, meaning the theoretical representation of the real world that goes beyond the observational data (ie, theoretical analysis).^44^ While statistical analysis is probably closer to the data, it is not necessarily closer to reality and empirical models can potentially lack firm theoretical grounding, not accounting for underlying system dynamics and unobserved but influential entities. On the other hand, theoretical models, are potentially speculative, untested and not fundamentally empirical. Therefore, statistical models need to move closer to theoretical models and vice versa. SM might serve as an example of a statistical model moving closer to a theoretical model. SM can unravel complex processes, temporal ordering, causal linkages and direct and indirect effects, despite being fairly statistical and empirical by only including variables that have been measured.^44^ ABMs might serve as an example of a theoretical model having moved closer to a statistical model, intermingling chance with determinism. In ABMs events occur either by chance, according to probability distributions or are governed by strong behavioral rules which are determinative and not subjective to chance. Nevertheless, in ABMs many elements remain beyond empirical observation and one has a theoretical model that is partly estimated statistically.^44^

 In line with Epstein’s thoughts that even the best models are *fruitfully wrong* and *illuminating abstractions,*^66^ one needs to recognize that models are limited systems, while they have the power to form the conceptual foundations of their respective fields and are *headlights in dark unexplored territory. *Models can surprise, make us curious, lead to new questions, allow us to doubt and thereby, help us to base our beliefs on evidence and not on authority.^66^

 As common in HIA studies, where evidence on causal inferences is lacking, due to a lack of supportive data or theory, researchers feed in assumptions on model parameter characteristics that carry uncertainties. Uncertainties in the model may be structural (ie, associated with model configuration) and parametric (ie, associated with model parameters, eg, slope of ERFs, threshold levels, etc). Uncertainties need to be transparently defined and quantified by giving ranges of confidence on plausible values. Uncertainty must be effectively communicated with the model design and results, and expectations about accuracy in forecasting of policy impacts must be managed.

 A final consideration is the model validation. For the majority of simpler models, a face-validity of the model (first order validation, ie, determination by experts that the model reflects the understanding of the available evidence and science) and an internal validation (second order validation, ie, verification of the adequacy of the codes and algorithms used for the modelling process) could be considered sufficient.^67^ While for some models, especially the ones with complex dynamics and feedbacks, a validation of third or higher order is recommended, which would be an external validation (third order validation, ie, model output are compared with empirical observation not considered in the model development), predictive validation (fourth order validation, ie, assessing model’s ability to predict empirical results that were not available during the model development) or a cross-model validation (ie, comparison of results among different models for the same or sufficiently similar analyses).^67^

###  Strengths and Limitations

 To our knowledge, this is the first narrative review comparing different mathematical models that have been or can be used for health impact forecasting of public policy proposals, and we provide key consideration for model choice for researchers who wish to engage in HIA modelling. The expert consultation and supportive narrative literature review add robustness to our findings. However, this is not a systematic review of HIA models nor relevant literature; therefore, we acknowledge that partially selective reporting, also of model examples, might have occurred. Moreover, as stated above, we recognize that models are often more integrated and cannot be distinguished as simply as we represent them here. We intend to describe basic model setup and functioning and acknowledge that working definitions in reality are much broader, hybrid-models exist, and distinguishing between model types as we do in this manuscript is often not possible.

## Conclusions

 We reviewed seven different models that can be used to forecast the health impacts of policy proposals, and therefore are relevant in the field of HIA. Each of these models has specific characteristics, related to available parameter and data structures, computational implementation, and comprehensibility, and the researcher should consider them critically before choosing a model. Model input assumptions need to match the available data structure, while the outputs of the modelling must match the research question to be answered and need to be comprehensible to the end-users the HIA is conducted for, while available resources (ie, time, costs, computational skills) are overarching and decisive. Moreover, good communication and transparency during the entire HIA process, from research question definition, model conception and implementation, to interpretation of modelling outputs, is needed to make HIA outcomes relevant to inform the policy-decision making process and generate impact for society.

## Acknowledgements

 We thank James Woodcock and Belen Zapata-Diomedi for their valuable comments on this manuscript. ISGlobal authors acknowledge support from the Spanish Ministry of Science and Innovation and State Research Agency through the “Centro de Excelencia Severo Ochoa 2019-2023” Program (CEX2018-000806-S), and support from the Generalitat de Catalunya through the CERCA Program.

## Ethical issues

 Not applicable.

## Competing interests

 Authors declare that they have no competing interests.

## Authors’ contributions

 NM and DR conceptualized the work, reviewed the literature, analyzed the data, drafted and revised the manuscript. RA, NB, HG, SJL, GdSM, APS, KG, CT, and MN reviewed the literature, provided expert input on the respective models and revised the manuscript. All authors approved the final manuscript.
